# Social determinants of health and cognition in a Panamanian sample of older adults

**DOI:** 10.1002/alz70857_102139

**Published:** 2025-12-24

**Authors:** Diana C Oviedo

**Affiliations:** ^1^ Instituto de Investigaciones Científicas y Servicios de Alta Tecnología (INDICASAT AIP), Centro de Neurociencias, Panamá, Panamá, Panama

## Abstract

**Background:**

Studies suggest that biological and social factors influence dementia risk. In healthy older women, social determinants of health such as age, sex, socioeconomic status, education, income, employment, and other health‐ related determinants like chronic illnesses, BMI, and depression contribute to maintenance or decline of cognitive function. To examine social and biological factors associated with cognitive variation in cognitively healthy older Panamanian women.

**Method:**

This longitudinal study analyzed cognitive domains at baseline (*n* = 357) and at a 17‐month follow‐up (*n* = 200) in women aged 60+ from the Panama Aging Research Initiative‐Health Disparities (PARI‐HD) study. Assessments included clinical questionnaires, physiological measures, and a neuropsychological test battery evaluating global cognition and seven cognitive domains. Multiple regression analyses assessed associations between demographic and clinical factors and baseline cognition, while repeated measures analyses examined cognitive changes over time.

**Result:**

Participants averaged 68.6 years of age (SD = 5.9) with 16.1 years of education (SD = 4.7). Almost half of participants (43.4%) reported subjective cognitive impairment. The total number of chronic illnesses, medications, and the prevalence of subjective cognitive decline increased at first follow‐up. Age, income, and education were strongly associated with baseline cognition. Subjective cognitive impairment correlated with lower global cognition, verbal learning, and memory performance. At follow‐up, only attention declined, with subjective health and depressive symptoms predicting this change.

**Conclusion:**

Studies have shown that in preclinical stages of AD, objective cognitive deficits are not present, nevertheless individuals may be noting subtle changes in their cognition that could be considered a risk factor of cognitive impairment. These findings contribute to understanding cognitive health in older Hispanic women and highlight sociodemographic and health‐related factors linked to cognitive decline and dementia risk.

